# “Time window” effect of Yoda1‐evoked Piezo1 channel activity during mouse skeletal muscle differentiation

**DOI:** 10.1111/apha.13702

**Published:** 2021-06-22

**Authors:** Alessandra Bosutti, Arthur Giniatullin, Yulia Odnoshivkina, Luca Giudice, Tarja Malm, Marina Sciancalepore, Rashid Giniatullin, Paola D'Andrea, Paola Lorenzon, Annalisa Bernareggi

**Affiliations:** ^1^ Department of Life Sciences University of Trieste Trieste Italy; ^2^ Department of Physiology Kazan State Medical University Kazan Russia; ^3^ Laboratory of Biophysics of Synaptic Processes Kazan Institute of Biochemistry and Biophysics Federal Research Center “Kazan Scientific Center of RAS” Kazan Russia; ^4^ A.I. Virtanen Institute for Molecular Sciences University of Eastern Finland Kuopio Finland; ^5^ B.R.A.I.N., University of Trieste Centre for Neuroscience Trieste Italy; ^6^ Institute of Fundamental Medicine and Biology Federal University Kazan Russia

**Keywords:** myogenesis, myotubes, Piezo1 channels, satellite cells, skeletal muscle myofibres, Yoda1

## Abstract

**Aim:**

Mechanosensitive Piezo1 ion channels emerged recently as important contributors to various vital functions including modulation of the blood supply to skeletal muscles. The specific Piezo1 channel agonist Yoda1 was shown to regulate the tone of blood vessels similarly to physical exercise. However, the direct role of Piezo1 channels in muscle function has been little studied so far. We therefore investigated the action of Yoda1 on the functional state of skeletal muscle precursors (satellite cells and myotubes) and on adult muscle fibres.

**Methods:**

Immunostaining, electrophysiological intracellular recordings and Ca^2+^ imaging experiments were performed to localize and assess the effect of the chemical activation of Piezo1 channels with Yoda1, on myogenic precursors, adult myofibres and at the adult neuromuscular junction.

**Results:**

Piezo1 channels were detected by immunostaining in satellite cells (SCs) and myotubes as well as in adult myofibres. In the skeletal muscle precursors, Yoda1 treatment stimulated the differentiation and cell fusion rather than the proliferation of SCs. Moreover, in myotubes, Yoda1 induced significant [Ca^2+^]*_i_* transients, without detectable [Ca^2+^]*_i_* response in adult myofibres. Furthermore, although expression of Piezo1 channels was detected around the muscle endplate region, Yoda1 application did not alter either the nerve‐evoked or spontaneous synaptic activity or muscle contractions in adult myofibres.

**Conclusion:**

Our data indicate that the chemical activation of Piezo1 channels specifically enhances the differentiation of skeletal muscle precursors, suggesting a possible new strategy to promote muscle regeneration.

## 1 INTRODUCTION

Piezo1 ion channels are Ca^2+^‐permeable non‐selective cation channels,^1^, ^2^ expressed in different cell types, where they regulate many functions such as stem cell differentiation,^3^ cell migration^4^ and exercise physiology.^5^

In blood vessel endothelial cells, Piezo1 has been identified as a key regulator of signalling pathways controlling vascular tone and blood pressure.^6^ Following Piezo1 channel activation, the endothelial membrane permeability to Na^+^ and Ca^2+^ increases, causing a dichotomous response. The induced membrane depolarization is responsible for vasoconstriction, which contrasts with the relaxation due to the endothelium‐derived hyperpolarization factor. The increase in endothelial Ca^2+^ influx induces the activation of nitric oxide synthase, responsible for vasodilation. By doing this, Piezo1 channels are proposed to be involved in redistribution of the blood flow during exercise, enhancing the performance of the skeletal muscle.^5^ Because their activation can mimic the benefits of physical exercise, Piezo1 channels have been recently defined as “exercise sensors.”^5^, ^7^ Consequently, Yoda1, the specific chemical activator of Piezo1 channels,^8^ has been suggested as a potential “exercise pill” able to overcome the negative consequences of muscle inactivity.^7^

Interestingly, Piezo1 channels are also reported in immature skeletal muscle cells, particularly in postnatal myogenic precursors.^1^, ^9^ Postnatal myogenesis, responsible for growth and muscle regeneration,^10^ is mediated by satellite cells (SCs), a quiescent myogenic stem cell population in intimate association with the plasma membrane of terminally differentiated myofibres.^11^ In response to specific stimuli, SCs are quickly activated to proliferate; some of them are committed to generate myoblasts that fuse to form multinucleated myotubes; the rest return uncommitted to replace the quiescent pool.^12^ Among the stimuli that promote the postnatal myogenesis, there are the mechanical stretches^13^, ^14^, ^15^ and muscle contractions,^16^, ^17^ both of which could potentially activate Piezo1 channels.

It is largely known that skeletal muscle cells express many types of mechanosensitive channels. However, because they can be activated by the same mechanical stimulus,^18^ the identification of the contribution of each of them has so far been difficult. Nowadays, the opportunity of a specific chemical activation of Piezo1 channels by Yoda1^8^ offers the novel advantage of overcoming such a limitation, allowing a better characterization of Piezo1 channel function in the context of skeletal muscle physiology. Recently, in the myogenic cell line C2C12, it has been reported that the expression of Piezo1 channels is important in controlling myoblast fusion into myotubes.^9^ Although these results are intriguing and the C2C12 cell line is widely used as a cell model to study myogenesis, the role of Piezo1 channels and the effect of Yoda1 on primary skeletal muscle cells presently remain unknown.

In this study, for the first time, we describe the effect of Yoda1 and chemically activated Piezo1 channels in a primary skeletal muscle cell model. To do this, experiments were performed on isolated mouse adult *flexor digitorum brevis* (*FDB*) myofibres,^17^, ^19^, ^20^ which offer a model to study the effect of cell treatments on terminally differentiated myofibres immediately after dissociation and in postnatal differentiating myogenic precursors in long‐term cell culture. In fact, when *FDB* myofibres are isolated and plated, the adherent SCs detach from them, proliferate, fuse with each other and develop myotubes recapitulating the main steps of the postnatal myogenesis.^17^ Specifically, our main goals were as follows: (a) to characterize the cellular localization of Piezo1 channels on primary SCs, myotubes and myofibres and (b) to test the effect of Yoda1‐mediated Piezo1 channel activity on key steps of the postnatal myogenesis and on adult myofibres.

## 2 RESULTS

### 2.1 Localization of Piezo1 channels

**FIGURE 1:**
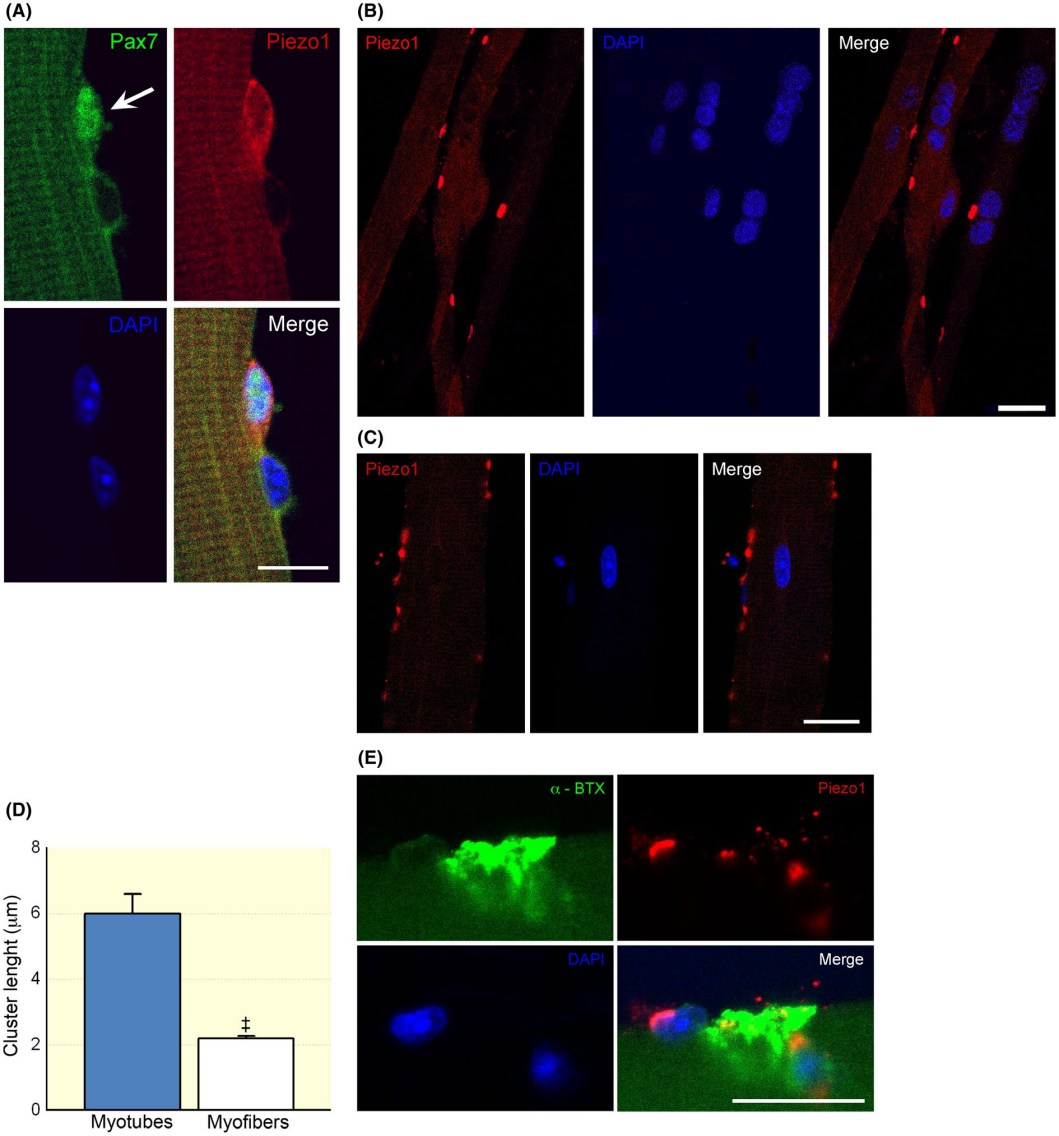
Localization of Piezo1 channels in skeletal muscle cells by confocal microscopy. A, Immunostaining for Piezo1 channels in a Pax7‐positive satellite cell (SC) (arrow) attached onto a *FDB* fibre (24 h post plating, 50 SCs were analysed). Note the close by Pax7‐negative nucleus likely belonging to the skeletal muscle fibre. B, Representative Piezo1 channel clusters detected in differentiating myotubes (7 day post plating). C, Immunostaining revealed Piezo1 channels organized in scattered clusters also in myofibres. Note the different scale bars in (B) and (C). D, Comparison of cluster length detected in myotubes and myofibres (n = 32 and 451 clusters analysed in myotubes and myofibres; ^‡^ *P* < .0001, Mann–Whitney test). E, Localization of Piezo1 channel clusters at a nearby endplate region of a myofibre (24 h post plating). Scale bars: 10 μm in (A) and 20 μm in (B), (C) and (E). Each group of confocal experiments were carried out on at least three independent cell culture preparations

Immunostaining experiments were performed to analyse the localization of Piezo1 channels on SCs, myotubes and myofibres in *FDB*‐derived cultures. Confocal analysis of immunolabelling for myogenic transcription factor Pax7 was used to identify quiescent and activated SCs.^21^ Piezo1 channel staining was detectable in Pax‐7 positive SCs that were still attached on the myofibre (Figure 1A). In multinucleated myotubes derived from SCs, the distribution of Piezo1 channels appeared as clusters after 7 days in culture (Figure 1B), similar to what was observed along the membrane of dissociated mouse myofibres analysed 24 hours after seeding (Figure 1C). In myotubes, however, the length of the Piezo1‐positive clusters was significantly greater than that found in myofibres (Figure 1D). Interestingly, in myofibres, we observed clusters of Piezo1 channels also nearby at the endplate region stained with Alexa‐488‐α‐bungarotoxin (Figure 1E). Antigen retrieval control experiments performed using the specific blocking peptide support the specificity of the Piezo1 antibodies in our cell model (Figure S1).

To confirm the expression of Piezo1 in mouse skeletal muscle cell precursors *in vivo*, we took advantage of the single‐cell sequencing data of hindlimbs of 3‐month‐old C56BL/6J mice^22^ to analyse Piezo1 coexpression across different cell populations in three different muscle stem cell types (muscular stem cell close to quiescence [MuSc cQ], muscular skeletal stem cell early activation [MuSc eA] and differentiated myocytes [DMs]). Clustering analysis revealed that all MuSc eA were both positive to Pax7, while DM cells were positive to myogenin (MyoG). Our data analysis showed that Piezo1 was more frequently found in MuSc cQ cells compared with 96.94% of all the other genes present in the same cell type, in MuSc eA cells the 92.29% and in MyoG‐positive myocytes the 77.38%, confirming an abundant expression of Piezo1 in muscle progenitors and myocytes also *in vivo* conditions. Moreover, the level of Piezo1 expression at the single‐cell level was also significantly high (MuSc cQ: 98.10% ± 0.075%, n = 119; MusSc eA: 94.35% ± 1.03%, n = 473; MyoG‐positive: 79.03% ± 5.12%, n = 183; Figure S2B). This highlights that the gene was needed strongly expressed in the groups.

### 2.2 Effect of Yoda1 on postnatal myogenesis

**FIGURE 2:**
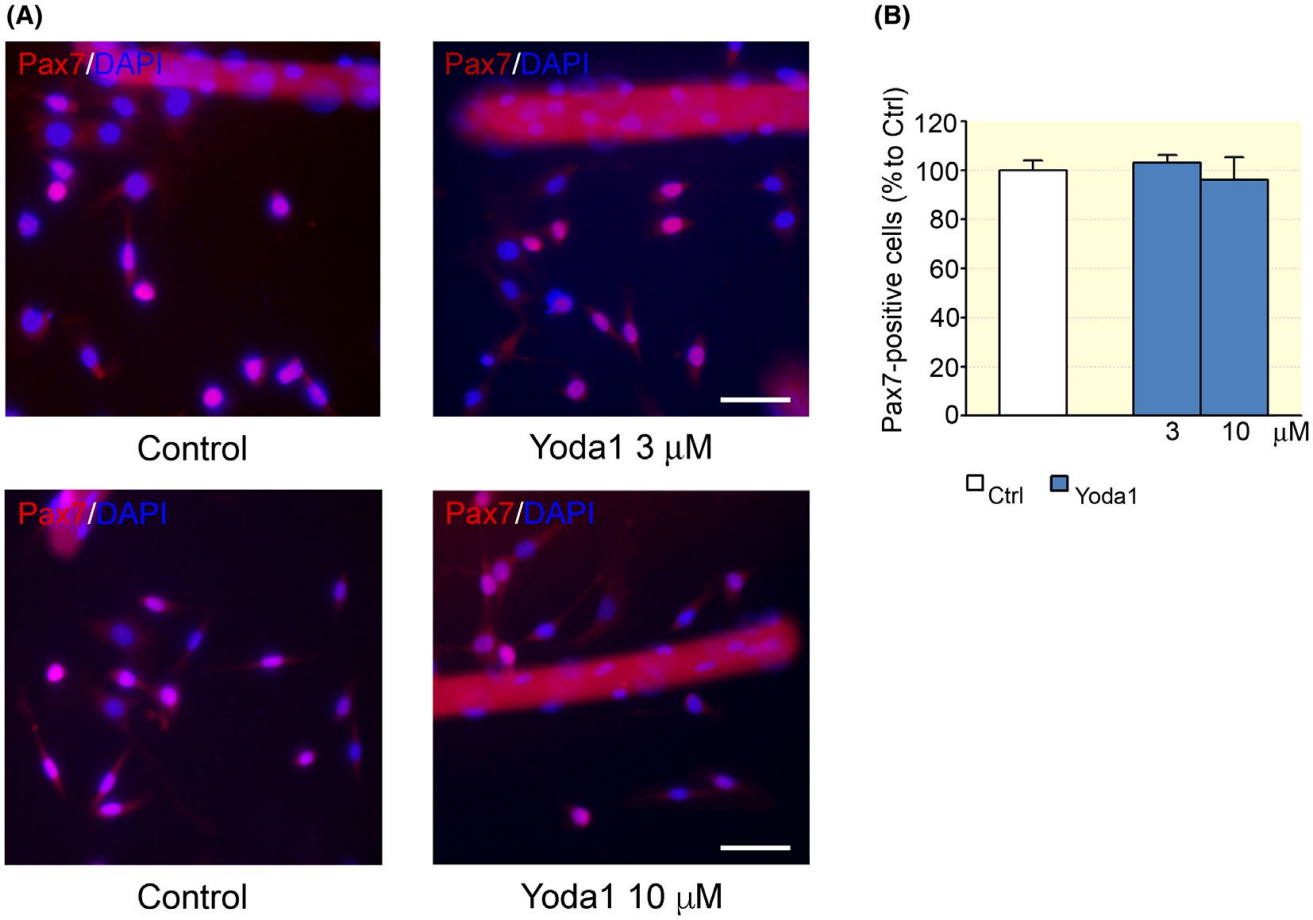
Effect of Yoda1 on Pax7‐positive cell numbers. A, Pax7‐positive satellite cells (SCs) after 48 h from the dissociation, in control conditions and in the presence of Yoda1 (3 and 10 μM). Scale bars: 50 μm. B, The cell treatment with 3 or 10 μM did not affect the percentage of Pax7‐positive cells (control: n = 131; 3 μM: n = 137; 10 μM: n = 29 optical fields; *P* > .05, ANOVA, Dunnett's post hoc test). Experimental replicates n = 4; data from five independent experiments

A set of experiments was aimed at investigating the possible effect of Piezo1 channel activity on SC number. For this purpose, the number of Pax7‐positive cells was analysed in *FDB* myofibre cultures treated with the agonist Yoda1 (3 and 10 μM) for 48 hours (Figure 2A). Within this time period, most of the cells are still uncommitted and the effect of Yoda1 on the proliferative phase of the myogenic precursors could be investigated.^17^ After 48‐hour treatment, the percentage of Pax7‐positive cells was similar to the one observed in untreated cultures (Figure 2B), thus ruling out major effects of chemically activated Piezo1 channels on SC proliferation and/or survival.

**FIGURE 3:**
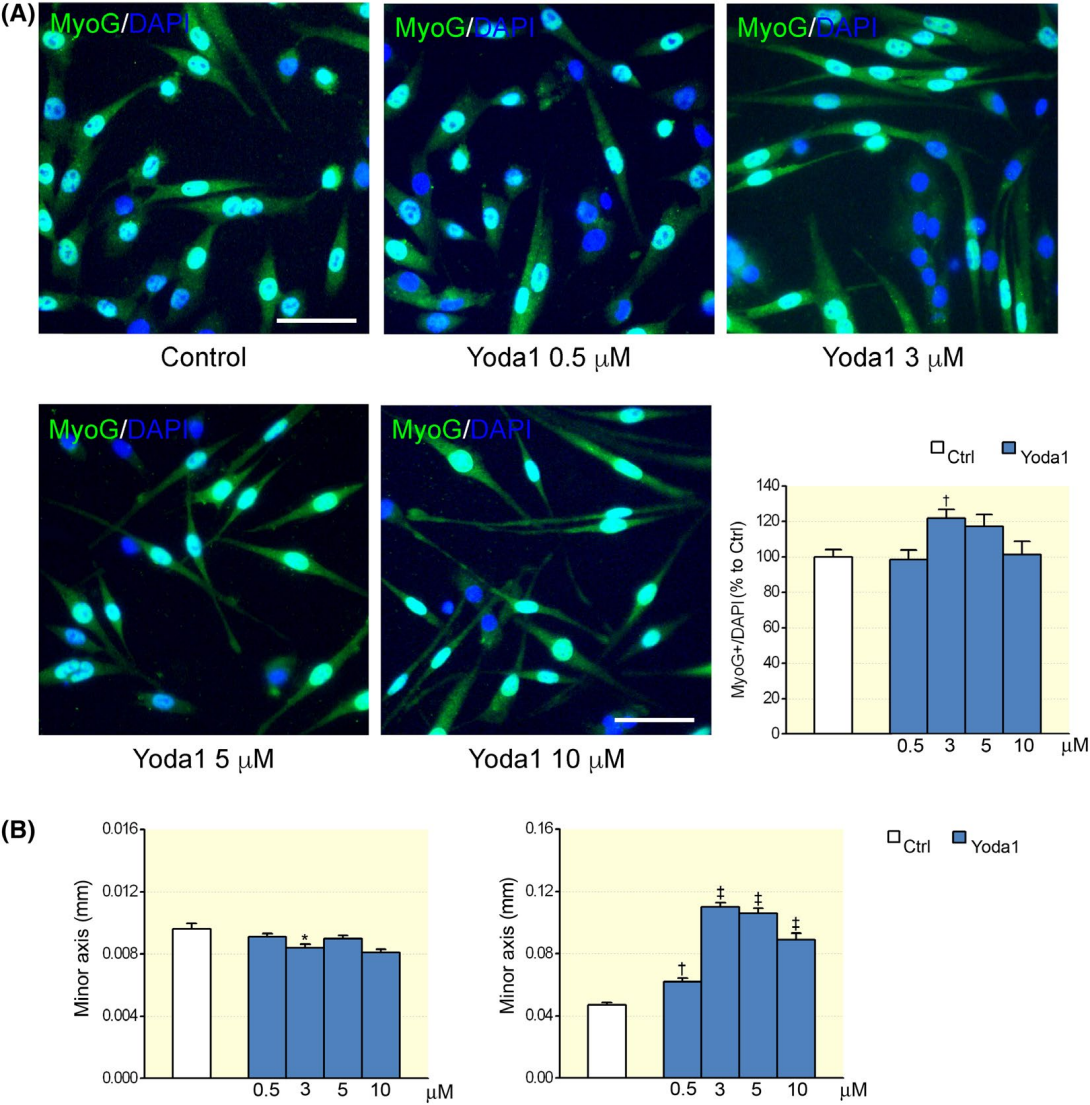
Dose–response effect of Yoda1 on myogenic precursors. A, Representative images and graph summarizing the percentage of MyoG‐positive cells cultured in differentiation medium for 72 h in controls and in the presence of different Yoda1 concentrations (control: n = 64 optical fields; 0.5 μM: n = 38; 3 μM: n = 62; 5 μM: n = 46; 10 μM: n = 37, ^†^ *P* < .01, ANOVA, Dunnett's post hoc test). B, Effect of Yoda1 on cell morphology (control: n = 192 optical fields; 0.5 μM: n = 185; 3 μM: n = 293; 5 μM: n = 244; 10 μM: n = 175, ^*^ *P* < .05, ^†^ *P* < .01 and ^‡^ *P* < .001, ANOVA, Dunnett's post hoc test) after a treatment of 72 h. Experimental replicates n = 2; data from two independent experiments

**FIGURE 4:**
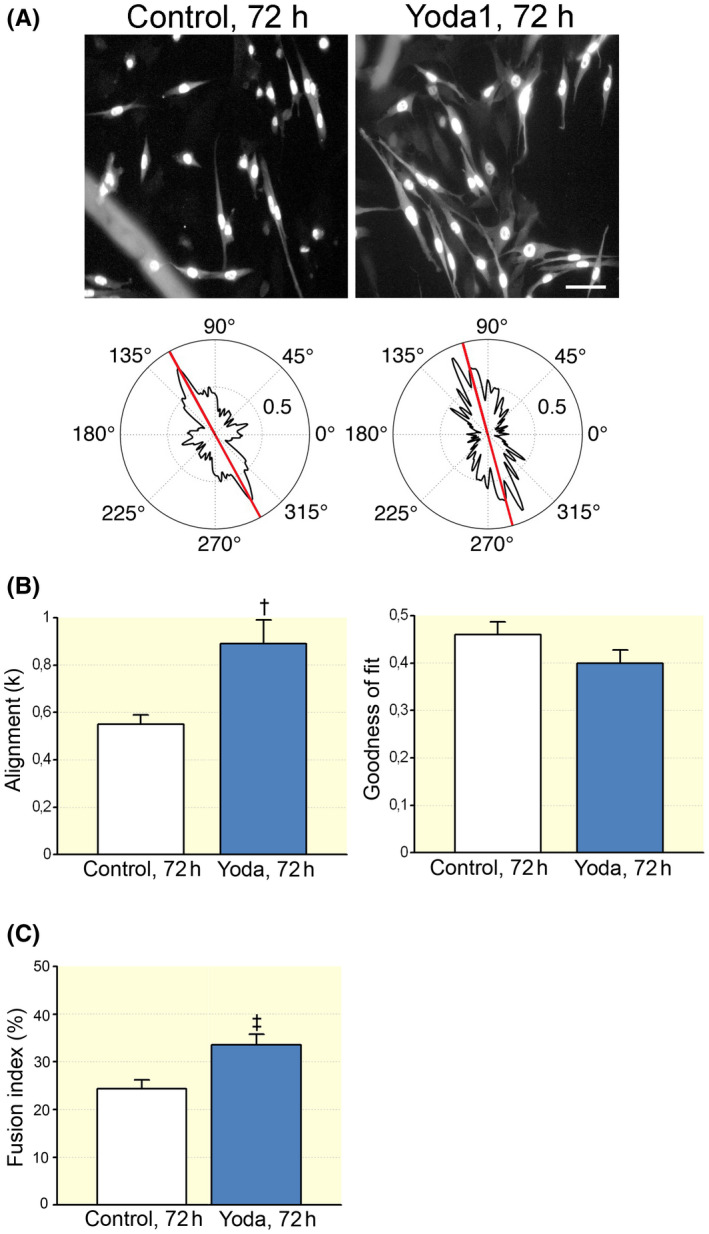
Yoda1 affected myocyte orientation. A, Representative 8‐bit greyscale images of MyoG‐positive cells cultured in differentiation medium for 72 h and, below, the corresponding analysis of the mean cell orientation using the FiberFit software. Scale bar: 50 μm. B, On the left, the dispersion parameter *k*, which quantifies the degree of cell alignment (for further details, see Section 4) in control condition (n = 69 cells) and in cells treated with Yoda1 (3 μM) (n = 61 cells; ^†^ *P* = .0089, *t* test). On the right, the goodness of fit *R* ^2^ (*P* = .14, Mann–Whitney test). Control and treated cells were from different culture dishes. Experimental replicates n = 3; data from two independent experiments. C, The presence of Yoda1 (3 μM) increased the number of multinucleated desmin‐positive cells (control: 24.38% ± 1.85%, n = 42 optical fields; Yoda1: 33.53% ± 2.28% , n = 53 optical fields; ^‡^ *P* = .0034, *t* test). Experimental replicates n = 3; data from four independent experiments

Then, we evaluated whether the activation of Piezo1 channels could play some role at a more advanced stage of myogenesis. To accomplish this aim, *FDB* myofibre cultures were exposed to different concentration of Yoda1, but the cultures were maintained in a differentiation medium, allowing the commitment and fusion of SCs into myotubes. In these experimental conditions, at first, immunostaining for MyoG was used to quantify SC differentiation into myocytes, the mononucleated myogenic precursors of myotubes^23^ (Figure 3A). We found that, after 72 hours of incubation at different concentration of the agonist (0.5, 3, 5 and 10 μM respectively), the fraction of MyoG‐positive myocytes was significantly increased at 3 μM. Moreover, as described for other cell types,^24^, ^25^ Yoda1 treatment caused cell elongation and myocytes visibly appeared longer as revealed by measuring minor and major cell axes; this effect was consistent for all concentration tested (Figure 3B). Intriguingly, at 3 μM point, Yoda1 also affected the orientation of cells (Figure 4A); treated cells tended to acquire a similar spatial orientation, appearing less dispersed and with a more similar alignment (Figure 4B). Taken together, these observations suggest that Piezo1 channels exert significant effect on different steps along myogenic differentiation.

This hypothesis was further supported by the observation that the Yoda1 treatment increased the fusion index of myogenic precursors (Figure 4C), indicating that the chemical activation of Piezo1 stimulates myotube formation (Figure S3). Yoda1 treatment did not affect the expression of desmin and myosin heavy chains detected in all the myotubes observed (Figure S4), indicating that the agonist did not impair myotube differentiation.

### 2.3 Effect of Yoda1 on adult myofibres

In adult *FDB* myofibres, as described, immunofluorescence revealed the presence of clusters of Piezo1 channels and some of them close to the endplate region (Figure 1E). Starting from this observation, we investigated whether Yoda1 modulated the synaptic activity at the adult NMJ.

**FIGURE 5:**
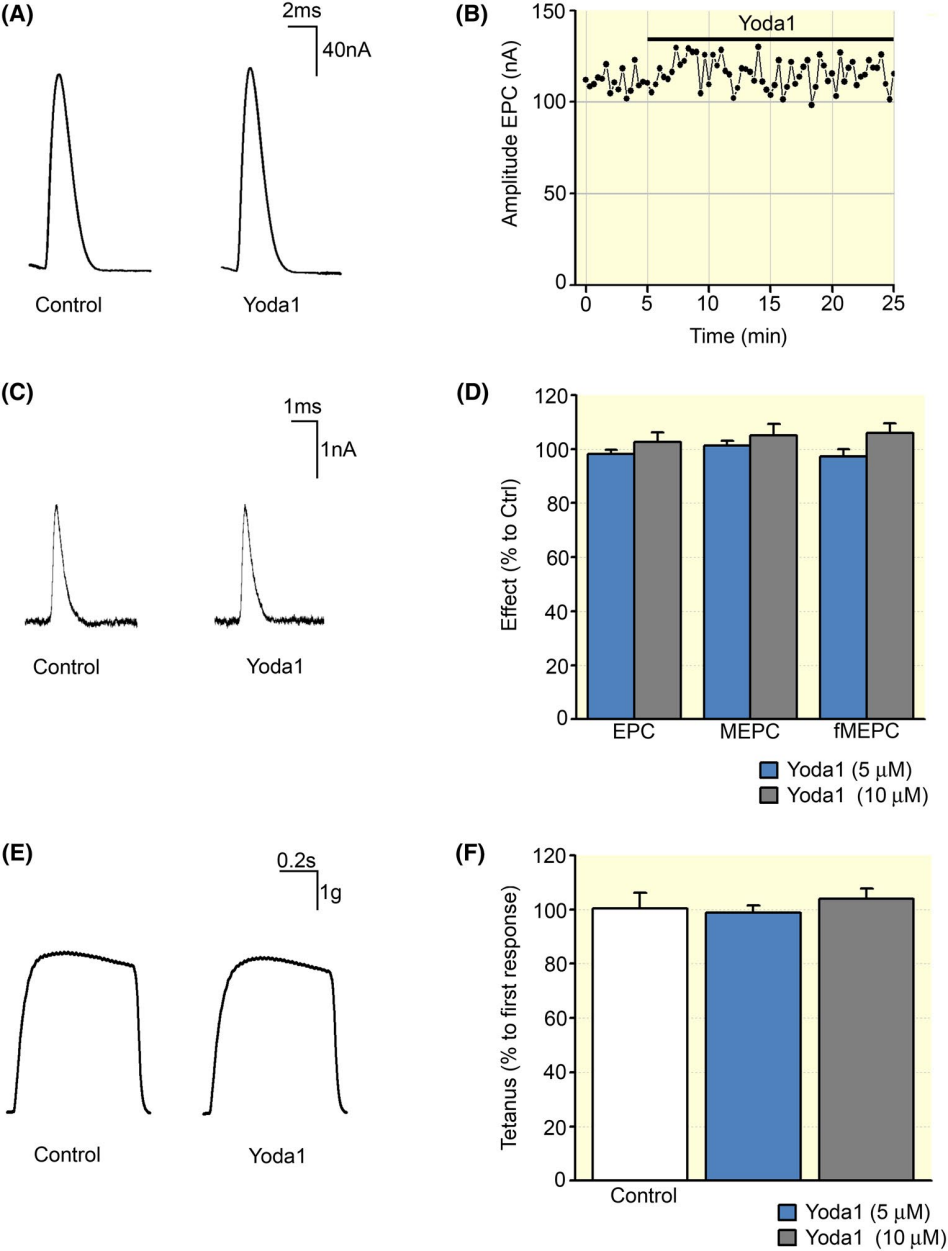
Effect of Yoda1 on adult skeletal muscles. A, Representative recording traces of multi quantal endplate currents (EPCs) in the absence (Control) and presence of Yoda1 (5 μM). B, The real dynamics of multi quantal EPC amplitude changes during recording in a separate neuromuscular junction. C, Representative recording traces of miniature endplate currents (MEPCs) in the absence (Control) and presence of Yoda1 (5 μM). D, Averages of the EPC and MEPC amplitudes and MEPC frequencies in absence and in presence of Yoda1 (5 and 10 μM) expressed as percentage respect to controls (Ctrl); n = 6 animals, paired *t* test. E, Example of tetanic contraction before and after Yoda1 application (5 μM). F, Averages of tetanus force recorded before and after Yoda1 (5 and 10 μM) expressed as percentage respect to controls (Ctrl); n = 6 animals, paired *t* test

**FIGURE 6:**
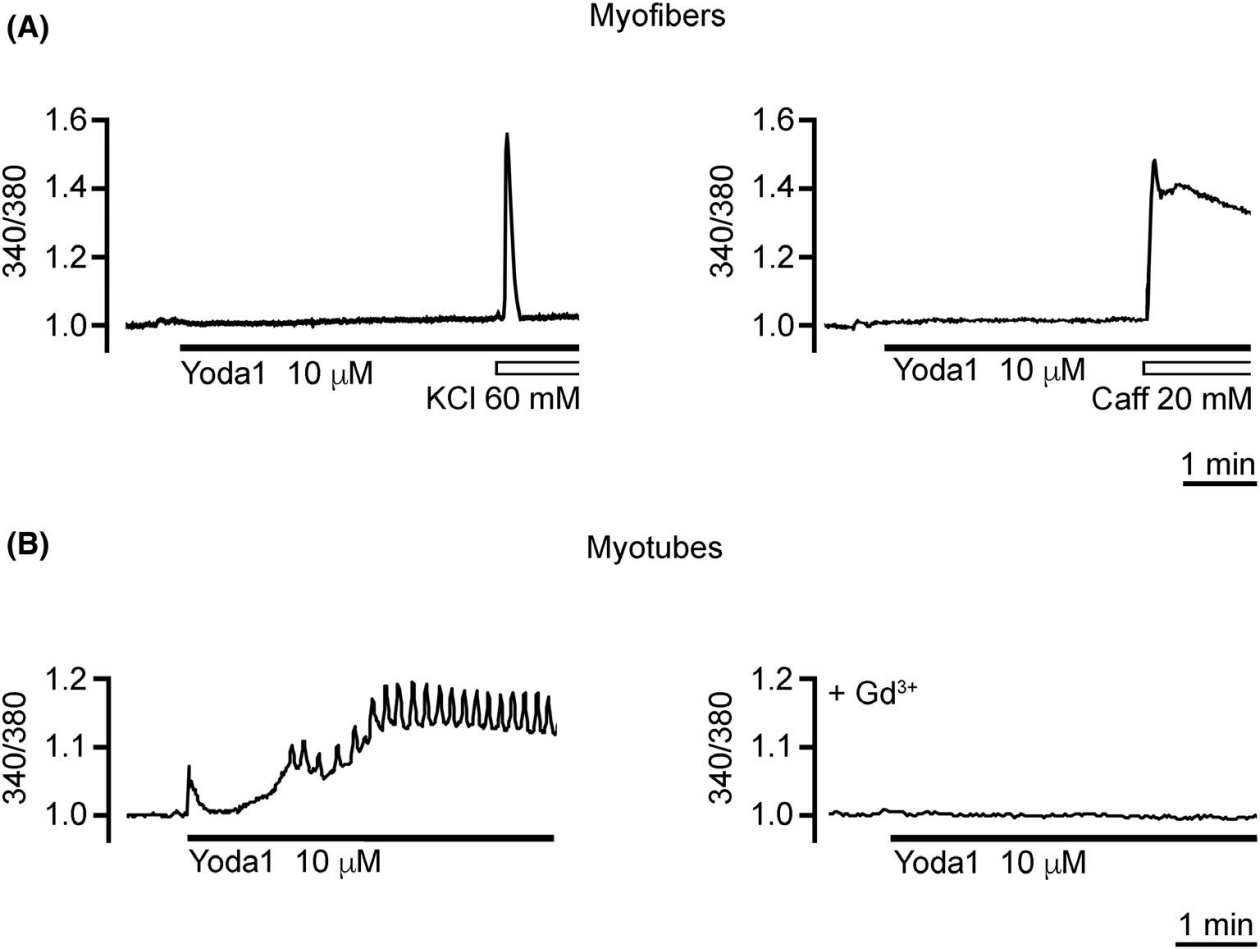
Effect of Yoda‐1 on [Ca^2+^]*_i_*. A, In *flexor digitorum brevis* (*FDB*) myofibres, Yoda1 (10 μM) did not alter the basal [Ca^2+^]*_i_*. The [Ca^2+^]*_i_* variations induced by high K^+^ depolarisation (KCl 60 mM) and caffeine (Caff 40 mM) were used as positive controls of cell responsiveness to stimuli able to increase the [Ca^2+^]*_i_*. B, In myotubes, at the same concentration, the agonist of Piezo1 channels induced [Ca^2+^]*_i_* oscillations in 35% of the observed myotubes (see text for further details). The effect of Yoda1 was prevented by myotube preincubation with Gd^3+^ (300 μM). Each set of Ca^2+^ imaging experiments was carried out on three independent cell culture preparations

A set of electrophysiological recordings was carried out on an acutely isolated mouse phrenic nerve‐diaphragm muscle preparation exposed to Yoda1 (5 and 10 μM) for 20 minutes. The mean amplitude of the endplate current (EPC) elicited by stimulating the motor nerve at 0.05 Hz was not changed during the treatment with Yoda1 (Figure 5A). In control conditions, the mean amplitude of the EPC was 123 ± 11 nA, and it remained stable after the exposure to 5 μM (n = 6) and 10 μM (n = 6) of the Piezo1 agonist (Figure 5B,D). Both concentrations did not alter neither the amplitude (MEPCs) nor the frequency of the miniature EPCs (fMEPCs, Figure 5C,D). In the set of experiments carried out at 5 μM, control MEPCs and fMEPCs were 2.1 ± 0.2 nA and 1.22 ± 0.7 s^−1^, respectively, while in the presence of the agonist, the MEPCs amplitude was 101.2% ± 1.8% and the frequency of the spontaneous events 97.0% ± 2.6% in respect to controls (Figure 5D). In the experiments conducted at 10 μM, control MEPCs and fMEPCs were 1.9 ± 0.3 nA and 0.98 ± 0.2 s^−1^ respectively and in the presence of the agonist 105% ± 4.2% and 106.0% ± 3.6% respect to controls (Figure 6D).

The effect of Yoda1 was also tested on the tetanic muscle contractions. The muscle response did not significantly change when a stimulation with the frequency of 50 Hz (0.3 ms, 30 pulses) was applied to the motor nerve after 20 minutes of incubation with the Piezo1 agonist (Figure 5E). The muscle response at 5 μM was 98% ± 3% (n = 6) and at 10 μM 103% ± 3% of their respective controls (n = 6, Figure 5F). In line with these results, the strength of muscle contractions remained unchanged when muscle contractions were induced at 0.1 Hz (by single current pulses causing the maximum contractile response) before and 20 minutes after 5 µM of Yoda1 application (98% ± 2%; n = 6; Figure S5).

In adult *FDB* myofibres, the clusters of Piezo1 channels appeared smaller than in myotubes (Figure 1D). Considering the Ca^2+^ permeability of Piezo1 channels,^1^ we used the live imaging technique to assess if the chemical activation of such small clusters could change the intracellular Ca^2+^ concentration ([Ca^2+^]*_i_*) in adult *FDB* myofibres. For this aim, Fura‐2‐loaded *FDB* myofibres were stimulated with 3 and 10 μM Yoda1.

In all the myofibres tested (n = 30), the Piezo1 agonist Yoda1 (3 and 10 μM) did not induce any Ca^2+^ response (Figure 6), whereas [Ca^2+^]*_i_* transients were always observed when KCl (60 mM) was used to depolarise the cell and activate the voltage‐operated Ca^2+^ channels or when caffeine (40 mM) was applied to induce Ca^2+^ release from intracellular stores.^26^ KCl and caffeine induced an increase in the basal [Ca^2+^]*_i_* of 58.83% ± 6.11% (n = 18) and 49.12% ± 8.16% (n = 12) respectively. In myotubes, 3 μM Yoda1 did not induce any detectable variations in [Ca^2+^]*_i_*, while 10 μM Yoda1 induced [Ca^2+^]*_i_* oscillations in ~35% of the observed cells (Figure 6; n = 34). The peak amplitude of the oscillations corresponded to an increase in the basal [Ca^2+^]*_i_* of 19.78% ± 0.82% (n = 12). The oscillation frequency varied from cell to cell in the range of 0.03‐0.40 Hz. The Yoda1‐evoked [Ca^2+^]*_i_* oscillations were invariably prevented when cells were pretreated with 300 μM Gd^3+^, a non‐selective blocker of mechanosensitive ion channels (Figure 6; n = 9). Taken together, these results suggest that in our experimental conditions, Yoda1 does not modulate any Ca^2+^ response in adult skeletal muscle fibres.

## 3 DISCUSSION

Piezo1 channels belong to the family of the mechanosensitive channels, particularly sensitive to fluid shear.^8^ In 2015, Yoda1 has been described for the first time as a key tool compound for studying the Piezo1 regulation and function.^8^ Thanks to its ability to mimic the effect of fluid shear stress,^5^ Yoda1 has been largely used to selectively activate the Piezo1 channels in many types of cells, such as endothelial cells,^2^ red blood cells,^27^ pancreatic acinar cells,^28^ trigeminal ganglia,^29^ precursors of skeletal muscle cells^9^ and many others, with limited non‐specific effects reported so far.^30^, ^31^ In this study, taking advantage of the availability of the agonist Yoda1, we investigated, for the first time, the effect of chemical activation of Piezo1 channels in primary skeletal muscle cells at different stages of myogenic differentiation, starting from SCs to myotubes and adult myofibres and the entire multicomponent NMJ. The main finding was that the chemical activation of Piezo1 channels exhibited its effect in a specific “time window” of the myogenic differentiation programme, favouring myogenic cell commitment and formation of myotubes.

Using mouse adult *FDB* myofibres isolated with their SC niches, we found that Piezo1 channels are present in myogenic precursors starting from a very early stage of myogenesis. Immunostaining revealed Piezo1 channels in quiescent and activated SCs (Pax7 positive) as already reported.^9^ In addition, we observed for the first time, the presence of Piezo1 channel clusters in myotubes and in adult skeletal *FDB* myofibres close to the endplate region. Interestingly, although detected from myogenic precursors up to terminally differentiated myofibres, the chemical activation of Piezo1 channels showed a stage‐specific enhancing effect on myogenic differentiation.

At least two reasons could explain the lack of a Yoda1 effect on SCs and adult myofibres even though Piezo1 was detectable by immunostaining. First, the lipidic composition of the cell membrane can change during myogenesis.^32^ The Piezo1 channel responds to forces within the membrane,^8^, ^33^, ^34^ and many studies reported the influence of lipids on the channel gating.^35^, ^36^, ^37^, ^38^ A transmembrane redistribution of phosphatidylserine has been reported to favour the activation of Piezo1 channels,^9^ and such redistribution does occur when the committed‐myogenic cells start to fuse into myotubes.^39^ Second, in the adult *FDB* myofibres, the absence of an effect on evoked or spontaneous synaptic transmission and muscle contractions even though Piezo1 channels were localized close to the endplate could be ascribed to the small size of the Piezo1 channel clusters. In line with this hypothesis, [Ca^2+^]*_i_* oscillations were observed in myotubes characterized by larger clusters. Interestingly, it has been already reported that the content of cholesterol in the cell membrane can affect Piezo1 channel clustering and the functional properties of the channels,^35^ confirming the importance of the composition of the cell membrane in determining the activity of Piezo1 channels at different stages of the myogenic differentiation programme.

Regarding the “time window” of efficacy of the chemical activation of Piezo1 channels, Yoda1 treatment induced two different effects: it favoured either the transition from a predominantly proliferative state to MyoG‐positive cells and their fusion into myotubes.

YAP and TAZ are two related transcriptional factors involved in the control of myogenic gene expression during the differentiation of the SCs.^40^ Interestingly, both of them have been recently proposed as downstream effectors of Piezo1 channel activity.^3^

Regarding the effect of Yoda1 on cell fusion into myotubes, it is known that myotube formation requires myocyte elongation, alignment and fusion. The myoblast elongation is a result of a deep organization of the actin filaments and microtubules^41^ and the remodelling of the actin cytoskeleton is essential for myoblast fusion.^42^ In the C2C12 cell line, the activity of Piezo1 channels was found to regulate the assembly of cortical actomyosin required for myotube formation,^9^ and a similar mechanism could occur in primary mouse myotubes. In addition, we observed that the Yoda1 effects on cell morphology and fusion were similar to those observed after the electrical pulse stimulation of *FDB* myofibres.^17^ In that case, the effect was mediated by the release of ATP from the contracting cells. Although still premature at this stage, a similar molecular mechanism could contribute to the chemical activation of Piezo1 channels. Interestingly, Piezo1 channels were found to induce the release of ATP in red blood cells,^43^ in endothelial cells and in mesenchymal stem cells where it controls cell migration.^44^

Finally, it should be noted that the chemical activation of Piezo1 channels may be different from the mechanical activation induced for instance by the physical exercise. Yoda1 may affect the sensitivity and the inactivation kinetics of Piezo1 by keeping the channels opened for long period of time.^8^ It is unknown if physical exercise causes a similar prolonged activation of the channels in vivo. A recent report demonstrates that, in endothelial cells, a specific local lipid environment is responsible for a noninactivating behaviour of Piezo1 channels, which enables endothelium to a sustained vascular flow sensing.^37^ A similar mechanism could be present also in skeletal muscle elements, which physiologically undergo to prolonged mechanical forces during the physical exercise.

In conclusion, our findings support the role for Piezo1 channels as the direct “exercise sensors.” Thus, the positive role of Piezo1 channels in the skeletal muscle is not limited to the increased blood supply^5^, ^6^ but has broader functions including the stimulatory effect on skeletal myogenesis. The possibility of chemical activation of Piezo1 channels within the specific time window suggests a new promising strategy to stimulate, in a stage‐specific manner, skeletal muscle plasticity, thereby increasing its regenerative capacity.

## 4 MATERIALS AND METHODS

### 4.1 *FDB* myofibre culture

FDB mouse myofibres with resistant SCs were isolated from 6‐ to 8‐week‐old C57/6J male mice as described in detail elsewhere.^17^ Animals were housed and sacrificed by cervical dislocation as approved by the local Animal Care Committee and by the European legislation (2010/63/EU). After enzymatic and mechanical dissociation, cells were plated on matrigel‐coated coverslips (1 mg/mL) and maintained in growth medium or differentiation medium according to the experimental purpose. The GM contained Dulbecco's modified Eagle's medium (DMEM) 64%, Medium 199 16%, foetal bovine serum (FBS) 20%, fetuin (25 μg/mL), bFGF (0.5 ng/mL), hEGF (5 ng/mL), insulin (5 μg/mL), dexamethasone (0.2 μg/mL) and gentamicin (50 μg/mL). The composition of DM was as follows: DMEM with high glucose, horse serum (5%), l‐glutamine (2 mM), penicillin (100 IU/mL) and streptomycin (100 μg/mL). Dishes were kept at 37℃ in saturated humidity and in CO_2_ (5%)‐enriched air. The culture medium was renewed every 48 hours. For the chronic treatment protocol, Yoda1 was added to the medium 2 hours after cell seeding. For each experimental point, the effect of the drug was compared with the untreated cells from the same animals (minimum number of animals = 3).

### 4.2 Immunostaining

FDB myofibres were fixed using 4% (w/v) paraformaldehyde in PBS (20 minutes; at 4℃). When needed, membrane permeabilization and blocking were performed using 5% normal goat serum in PBS/0.1% Triton‐X100 (30‐minute incubation).

In the set of experiments carried out to compare the cluster length of Piezo1 channels, immunostaining was performed skipping the cell membrane permeabilization. According to the experimental aims, the staining was performed by overnight incubation at 4℃ in normal goat serum with primary mouse monoclonal anti‐Pax7 (1:8 dilution), anti‐MyoG (1:20) or desmin (1:50); rabbit polyclonal anti‐PIEZO‐1 (1:50, NBP1‐78446) (1:8 dilution), anti‐MyoG (1:20), desmin (1:50) or anti‐Myosin Heavy Chain (1:20). For antigen retrieval experiments, Piezo1 antibody was preabsorbed overnight (at 4℃) by a 10‐fold excess of Piezo1 antibody blocking peptide with conditions as provided by the manufacturer. After incubation with the primary antibodies and washout with PBS/0.1% Triton‐X100, coverslips were incubated for 1.5 hours at RT with the secondary antibodies Alexa Fluor 594 goat anti‐mouse IgG, Alexa Fluor 568 goat anti‐rabbit IgG, Alexa Fluor 488 anti‐mouse or anti‐rabbit IgG. Nuclei were counterstained by 4′,6‐diamidino‐2‐phenylindole (DAPI, 1:50). Finally, cells were washed three times with PBS/0.1% Triton‐X100 and then mounted onto slides. Only dishes containing a comparable number of myofibres (on average from 100 to 150 per dish) were used for immunofluorescence experiments. When appropriate, in freshly dissociated myofibres, the endplate region was stained with Alexa‐488‐α‐bungarotoxin (2.5 μg/mL in PBS supplemented with 0.1% bovine serum albumin [BSA], 1 hour at room temperature).

Epifluorescence microscopy images were visualized by a Nikon Eclipse E800 microscope (Nikon Corporation). Images were captured with a Nikon DXM1200 digital camera. Image analysis was performed by Fiji‐ImageJ. Sizing, cropping and overlays of the images were conducted using Adobe Photoshop CC (Adobe Systems Incorporated). For the SC counting, we considered the Pax7‐positive cells both adherent to and migrated from the fibres and those that migrate from them.

Confocal microscopy images were captured with a Nikon C1si confocal microscope equipped with an argon laser (457, 477, 488, and 514 nm lines), a 561‐ and a 640‐nm diode laser and a Plan‐Apochromat 60×/1.4 (NA) oil‐immersion objectives. The spatial resolution was set at 100 nm. A series of optical images was collected at 0.30‐μm *z* resolution by sequential line scanning to avoid potential cross‐talk phenomena among fluorophores. Images were acquired keeping the same acquisition settings for laser intensity, pmt amplification, pinhole aperture and pixel dwell in order to obtain comparable signals between control and treated conditions. Confocal images were processed for *z* projection in maximum intensity by Fiji‐ImageJ. In the confocal images analysed for the quantification of Piezo1 cluster length, fluorescent signals above the threshold were considered clusters only if their longest axes were at least 2 μm.

### 4.3 Cell orientation, dispersion and fusion index

The cell morphology analysis was measured in cells expressing MyoG‐positive nuclei after 72 hours of culture in differentiation medium. The major and minor cell axes were measured in bright field microscopy using Fiji‐ImageJ.^17^

Cell orientation and dispersion analysis were carried out on epifluorescence images converted into 8‐bit greyscale and processed in two‐dimensional images at a single size (1024 × 1024 pixels) by Fiji‐ImageJ. Processed images were than analysed using FiberFit^45^, ^46^ (http://coen.boisestate.edu/ntm/fiberfit/) to generate a cell orientation distribution, to calculate the degree of fibre alignment *k* (where low *k* values correspond to disordered networks and large *k* values to aligned networks) and the goodness of the fit (*R* ^2^) to a von Mises distribution.

Experiments were conducted on at least three independent cell culture preparations. For each experimental point, at least 30 optical fields and 300 myogenic cells were examined.

The fusion index was established by dividing the number of nuclei in the myotubes (ie, cells having more than two nuclei) by the total number of nuclei observed in 50 fields randomly chosen.^47^

### 4.4 Ca^2+^ imaging

The [Ca^2+^]*_i_* was measured using Fura‐2 pentacetoxymethyl ester (Fura‐2 AM). Cell loading and image acquisition were carried out as described in detail elsewhere.^17^

In the temporal plots of the [Ca^2+^]*_i_* variations, the fluorescence ratio at rest was assumed to be 1. For the analysis, the amplitude of the single transients or oscillations was calculated as percentage of increase (measured at peak) versus basal [Ca^2+^]*_i_* value. The frequency of [Ca^2+^]*_i_* oscillations (in Hz) was calculated dividing the number of oscillation peaks by the recording time.

### 4.5 Electrophysiological recordings

The electrophysiological recordings were performed on half of the diaphragm muscle of outbred B6/SJL mice (22‐25 g) of both sexes. The animals were maintained in a 12 hour light/12 hour dark cycle with free access to food and water and then sacrificed in agreement with local Animal Care Committee and European legislation (2010/63/EU). Diaphragm muscle supplied with the phrenic nerve was isolated and then attached to the bottom of a Sylgard‐lined chamber, which was superfused at 2 mL/min throughout the experiment with physiological solution containing (in mM): NaCl 120, KCl 5, CaCl_2_ 2, MgCl_2_ 1, NaH_2_PO_4_ 1, NaHCO_3_ 24 and glucose 11. Solutions were saturated with a 5% CO_2_ and 95% O_2_ mixture, and pH was adjusted to 7.4 with NaOH/HCl. Experiments were performed at 24–25℃, which allows the muscle to maintain a stable level of neurotransmitter release for a long period.^48^

Postsynaptic EPCs and MEPCs recordings were performed using intracellular glass microelectrodes (tip diameter ~1 µm, resistance 3‐5 MΩ, filled with 2.5 M KCl) and the two‐electrode voltage‐clamp technique, as previously described.^48^, ^49^, ^50^ To maintain the physiological level of quantal release and avoid contractions^51^ during the EPC recordings, the muscle fibres were transversely cut (“cut muscles”). In this case, the *V*_h_ (holding potential) was kept at −40 mV and the recording started after the stabilization of the cell membrane potential (in about 40 minutes), as described elsewhere.^52^ EPCs were elicited by a single supramaximal phrenic nerve stimulation (0.05 Hz, 0.1‐ms duration) via a suction electrode connected to an extracellular stimulator (DS3 Digitimer Ltd.). The recorded EPCs and MEPCs were analysed offline using a PC and a custom‐made software.^49^ For the experiments on uncut muscles, the MEPCs were recorded by keeping the membrane potential of the fibres at −60 mV.

### 4.6 Recording of isometric contractions

For muscle contraction measurements, the left and right diaphragm were suspended under a constant tension of 5 g in a 20‐mL organ bath (15 mL/min perfusion rate) containing aerated (5% CO_2_ and 95% O_2_) physiological solution (pH 7.4, 25℃). Supramaximal pulses (0.1 Hz, 0.3 millisecond, 3‐6 V) and tetanic stimulation (50 Hz, 0.3 millisecond, 30 pulses) delivered by a DS3 Digitimer Ltd., were applied by electrodes placed on the motor nerve. Isometric muscle tension was recorded using a force transducer (MLT0420; AD Instruments). One end of the diaphragm muscle (lower edge) was tied to a fixed nail, and the other end was linked to a force transducer. A constant passive tension was kept. The preparations were allowed to stabilize for at least 20 minutes before onset of drug applications (Power‐Lab installation, AD Instruments). The signals were recorded and analysed using LabChart Pro software.

### 4.7 Trascriptome analysis of Piezo1 expression

The analysis of Piezo1 expression was obtained by the transcriptome data of Dell'Orso et al^22^ performed on muscle SCs (MuSCs) and primary myoblasts (PM) obtained from homeostatic or regenerating muscles by single‐cell RNA sequencing. The analysis was replicated as described in the section “Data processing and clustering” of Dell'Orso et al^22^ to extrapolate the single‐cell populations identified by the authors, specifically the Pax7‐positive cell populations named MuSc cQ and MuSc eA and the MyoG‐positive population (DMs).

The genes were captured with the sequencing in term of frequency and expression in each cell type‐specific population. First, the cells expressing a particular gene were counted and genes ranked based on their frequency (eg, Rank 1 for the gene expressed in the lowest number of cells). Then, they were divided by the number of total genes to scale the ranks from 0 to 1 (ie, the highest rank). The resulting value (ie, ratio) indicated how much the number of cells expressing a gene was high with respect the frequency of all the other genes in the same specific cell type (eg, the gene that appears expressed in the lowest number of cells gets a ratio close to 0, while the gene with the highest frequency gets a ratio equal to 1). The same approach was used to compare the expression value of the genes by a particular cell; because a gene was expressed in more than one cell of a population, the gene's expression ratio for every type‐specific cell was retrieved. The expression ratios referring to one gene indicate how much its expression levels were high with respect the expression of all the other genes in the same cells.

### 4.8 Chemicals

DMEM (high glucose) and horse serum were from Sigma‐Aldrich; FBS was from Gibco; l‐glutamine, penicillin, trypsin and streptomycin were from Euroclone; matrigel was from Corning; and gentamycin, fetuin, hEGF, bFGF and trypsin were from Life Technologies. Insulin and dexamethasone, Yoda1, gadolinium and Fura‐2 AM were from Sigma‐Aldrich; Medium 199, gentamycin, fetuin, hEGF and bFGF were from Life Technologies; and Alexa‐488‐α‐bungarotoxin was from Invitrogen.

### 4.9 Antibodies

Rabbit polyclonal anti‐Piezo1 affinity purified antibody (NBP1‐78446SS) and Piezo1 antibody blocking peptide (NBP1‐78446PE) were from NOVUS Biologicals; mouse monoclonal anti‐Pax7 MAB1675 was from R&D Systems; monoclonal mouse anti‐human desmin (clone D33, M076001‐2) antibody was from DAKO; mouse monoclonal anti‐MyoG (5FD, sc‐52903) and rabbit polyclonal anti‐Myosin Heavy Chain (H‐300) were from Santa Cruz Biotechnology; Alexa Fluor 594 goat anti‐mouse IgG and Alexa Fluor 488 goat anti‐mouse and anti‐rabbit IgG were from Jackson ImmunoResearch Laboratories; and Alexa Fluor 568 goat anti‐rabbit IgG was from Invitrogen.

### 4.10 Statistics

GraphPad Prism 4.00 (GraphPadSoftware) was used to analyse the data. To determine whether sample data were drawn from a Gaussian distributed population, a normality test was chosen. In case of parametric data, a *t* test or an ANOVA test (with Dunnett's post hoc test) for multiple comparisons was used for the statistical significance. For nonparametric data, a Mann–Whitney or Kruskall–Wallis test (with Dunn's post hoc test) for multiple comparisons was performed. The mean ± standard error (SEM) was used to compare the results, with a *P* < .05 for the statistical significance.

## CONFLICT OF INTEREST

The authors declare no conflict of interest.

## Supporting information

Fig S1‐S5

## References

[1] Coste B , Mathur J , Schmidt M , et al. Piezo1 and Piezo2 are essential components of distinct mechanically activated cation channels. Science. 2010;330(6000):55‐60. 20813920 10.1126/science.1193270 PMC3062430

[2] Wang Y , Xiao B . The mechanosensitive Piezo1 channel: structural features and molecular bases underlying its ion permeation and mechanotransduction. J Physiol. 2018;596(6):969‐978. 29171028 10.1113/JP274404 PMC5851880

[3] Pathak MM , Nourse JL , Tran T , et al. Stretch‐activated ion channel Piezo1 directs lineage choice in human neural stem cells. Proc Natl Acad Sci USA. 2014;111(45):16148‐16153. 25349416 10.1073/pnas.1409802111 PMC4234578

[4] Hung WC , Yang JR , Yankaskas CL , et al. Confinement sensing and signal optimization via Piezo1/PKA and myosin II pathways. Cell Rep. 2016;15(7):1430‐1441. 27160899 10.1016/j.celrep.2016.04.035 PMC5341576

[5] Rode B , Shi J , Endesh N , et al. Piezo1 channels sense whole body physical activity to reset cardiovascular homeostasis and enhance performance. Nat Commun. 2017;8(1):350. 10.1038/s41467-017-00429-3 28839146 PMC5571199

[6] Allison SJ . Hypertension: mechanosensation by PIEZO1 in blood pressure control. Nat Rev Nephrol. 2017;13(1):3. 10.1038/nrneph.2016.165 27840417

[7] Beech DJ . Endothelial Piezo1 channels as sensors of exercise. J Physiol. 2018;596(6):979‐984. 29194632 10.1113/JP274396 PMC5851887

[8] Syeda R , Xu J , Dubin AE . Chemical activation of the mechanotransduction channel Piezo1. eLife. 2015;4 10.7554/elife.07369. PMC4456433 26001275

[9] Tsuchiya M , Hara Y , Okuda M , et al. Cell surface flip‐flop of phosphatidylserine is critical for PIEZO1‐mediated myotube formation. Nat Commun. 2018;9(1):2049. 10.1038/s41467-018-04436-w 29799007 PMC5967302

[10] Zammit PS , Heslop L , Hudon V , et al. Kinetics of myoblast proliferation show that resident satellite cells are competent to fully regenerate skeletal muscle fibers. Exp Cell Res. 2002;281(1):39‐49. 12441128 10.1006/excr.2002.5653

[11] Mauro A . Satellite cell of skeletal muscle fibers. J Biophys Biochem Cytol. 1961;9(2):493‐495. 13768451 10.1083/jcb.9.2.493 PMC2225012

[12] Chang NC , Rudnicki MA . Satellite cells: the architects of skeletal muscle. In: Rendl M, ed. Current Topics in Developmental Biology (pp. 161‐181). Amsterdam: Elsevier; 2014. 10.1016/B978-0-12-416022-4.00006-8 24439806

[13] Tatsumi R , Hattori A , Ikeuchi Y , Anderson JE , Allen RE . Release of hepatocyte growth factor from mechanically stretched skeletal muscle satellite cells and role of pH and nitric oxide. Mol Biol Cell. 2002;13(8):2909‐2918. 12181355 10.1091/mbc.E02-01-0062 PMC117951

[14] Wozniak AC , Pilipowicz O , Yablonka‐Reuveni Z , et al. C‐met expression and mechanical activation of satellite cells on cultured muscle fibers. J Histochem Cytochem. 2003;51(11):1437‐1445. 14566016 10.1177/002215540305101104 PMC3957553

[15] Hara M , Tabata K , Suzuki T , et al. Calcium influx through a possible coupling of cation channels impacts skeletal muscle satellite cell activation in response to mechanical stretch. Am J Physiol Cell Physiol. 2012;302(12): C1741‐C1750. 10.1152/ajpcell.00068.2012 22460715

[16] Bazgir B , Fathi R , Valojerdi MR , Mozdziak P , Asgari A . Satellite cells contribution to exercise mediated muscle hypertrophy and repair. Cell J. 2016;18(4):473‐484. 28042532 10.22074/cellj.2016.4714 PMC5086326

[17] Bosutti A , Bernareggi A , Massaria G , et al. A “noisy” electrical stimulation protocol favors muscle regeneration in vitro through release of endogenous ATP. Exp Cell Res. 2019;381(1): 121‐128. 10.1016/j.yexcr.2019.05.012 31082374

[18] Stiber JA , Seth M , Rosenberg PB . Mechanosensitive channels in striated muscle and the cardiovascular system: not quite a stretch anymore. J Cardiovasc Pharmacol. 2009;54(2):116‐122. 19597371 10.1097/FJC.0b013e3181aa233f PMC4280092

[19] Grohovaz F , Lorenzon P , Ruzzier F , Zorec R . Properties of acetylcholine receptors in adult rat skeletal muscle fibers in culture. J Membr Biol. 1993;136(1):31‐42. 8271271 10.1007/BF00241487

[20] Rosenblatt JD , Lunt AI , Parry DJ , Partridge TA . Culturing satellite cells from living single muscle fiber explants. Vitr Cell Dev Biol Anim. 1995;31(10):773‐779. 10.1007/BF02634119 8564066

[21] Cornelison DDW , Wold BJ . Single‐cell analysis of regulatory gene expression in quiescent and activated mouse skeletal muscle satellite cells. Dev Biol. 1997;191(2):270‐283. 9398440 10.1006/dbio.1997.8721

[22] Dell'Orso S , Juan AH , Ko K‐D , et al. Single‐cell analysis of adult skeletal muscle stem cells in homeostatic and regenerative conditions. Development. 2019;146(12):dev174177. 10.1242/dev.174177 30890574 PMC6602351

[23] Brun CE , Wang YX , Rudnicki MA . Single EDL myofiber isolation for analyses of quiescent and activated muscle stem cells. In: Walker JM, ed. Methods in Molecular Biology (pp. 149‐159). Switzerland: Humana Press Inc.; 2018. 10.1007/978-1-4939-7371-2_11 29030819

[24] Chubinskiy‐Nadezhdin VI , Vasileva VY , Vassilieva IO , Sudarikova AV , Morachevskaya EA , Negulyaev YA . Agonist‐induced Piezo1 activation suppresses migration of transformed fibroblasts. Biochem Biophys Res Commun. 2019;514(1):173‐179. 31029419 10.1016/j.bbrc.2019.04.139

[25] Jetta D , Bahrani Fard MR , Sachs F , Munechika K , Hua SZ . Adherent cell remodeling on micropatterns is modulated by Piezo1 channels. Sci Rep. 2021;11(1):5088. 10.1038/s41598-021-84427-y 33658557 PMC7930019

[26] Lorenzon P , Bandi E , De Guarrini F , et al. Ageing affects the differentiation potential of human myoblasts. Exp Gerontol. 2004;39(10):1545‐1554. 15501025 10.1016/j.exger.2004.07.008

[27] Cahalan SM , Lukacs V , Ranade SS , Chien S , Bandell M , Patapoutian A . Piezo1 links mechanical forces to red blood cell volume. Elife. 2015;4(May):e07370. 10.7554/eLife.07370 PMC4456639 26001274

[28] Romac JMJ , Shahid RA , Swain SM , Vigna SR , Liddle RA . Piezo1 is a mechanically activated ion channel and mediates pressure induced pancreatitis. Nat Commun. 2018;9(1):1715. 10.1038/s41467-018-04194-9 29712913 PMC5928090

[29] Della PA , Mikhailov N , Giniatullin R . The emerging role of mechanosensitive piezo channels in migraine pain. Int J Mol Sci. 2020;21(3):696. 10.3390/ijms21030696 PMC7037473 31973098

[30] dela Paz NG , Frangos JA . Yoda1‐induced phosphorylation of Akt and ERK1/2 does not require Piezo1 activation. Biochem Biophys Res Commun. 2018;497(1):220‐225. 29428723 10.1016/j.bbrc.2018.02.058 PMC5835220

[31] Yoneda M , Suzuki H , Hatano N , et al. PIEZO1 and TRPV4, which are distinct mechano‐sensors in the osteoblastic MC3T3‐E1 cells, modify cell‐proliferation. Int J Mol Sci. 2019;20(19):4960. 10.3390/ijms20194960 PMC6801562 31597314

[32] Briolay A , Jaafar R , Nemoz G , Bessueille L . Myogenic differentiation and lipid‐raft composition of L6 skeletal muscle cells are modulated by PUFAs. Biochim Biophys Acta Biomembr. 2013;1828(2):602‐613. 10.1016/j.bbamem.2012.10.006 23079583

[33] Lewis AH , Grandl J . Mechanical sensitivity of Piezo1 ion channels can be tuned by cellular membrane tension. Elife. 2015;4:e12088. 10.7554/eLife.12088 26646186 PMC4718726

[34] Cox CD , Bae C , Ziegler L , et al. Removal of the mechanoprotective influence of the cytoskeleton reveals PIEZO1 is gated by bilayer tension. Nat Commun. 2016;7:10366. 10.1038/ncomms10366 26785635 PMC4735864

[35] Ridone P , Pandzic E , Vassalli M , et al. Disruption of membrane cholesterol organization impairs the activity of PIEZO1 channel clusters. J Gen Physiol. 2020;152(8):e201912515. 10.1085/jgp.201912515 32582958 PMC7398139

[36] Romero LO , Massey AE , Mata‐Daboin AD , et al. Dietary fatty acids fine‐tune Piezo1 mechanical response. Nat Commun. 2019;10(1):1200. 10.1038/s41467-019-09055-7 30867417 PMC6416271

[37] Shi J , Hyman AJ , De Vecchis D , et al. Sphingomyelinase disables inactivation in endogenous PIEZO1 channels. Cell Rep. 2020;33(1):108225. 10.1016/j.celrep.2020.108225 33027663 PMC7539531

[38] Buyan A , Cox CD , Barnoud J , et al. Piezo1 forms specific, functionally important interactions with phosphoinositides and cholesterol. Biophys J. 2020;119(8):1683‐1697. 32949489 10.1016/j.bpj.2020.07.043 PMC7642233

[39] Van den Eijnde SM , Van den Hoff MJB , Reutelingsperger CPM , et al. Transient expression of phosphatidylserine at cell‐cell contact areas is required for myotube formation. J Cell Sci. 2001;114(20):3631‐3642. 11707515 10.1242/jcs.114.20.3631

[40] Tao H , Zhu M , Lau K , et al. Oscillatory cortical forces promote three dimensional cell intercalations that shape the murine mandibular arch. Nat Commun. 2019;10(1):1703. 10.1038/s41467-019-09540-z 30979871 PMC6461694

[41] Tassin AM , Maro B , Bornens M . Fate of microtubule‐organizing centers during myogenesis in vitro. J Cell Biol. 1985;100(1):35‐46. 3880758 10.1083/jcb.100.1.35 PMC2113478

[42] Swailes NT , Knight PJ , Peckham M . Actin filament organization in aligned prefusion myoblasts. J Anat. 2004;205(5):381‐391. 15575887 10.1111/j.0021-8782.2004.00341.x PMC1571354

[43] Cinar E , Zhou S , Decourcey J , Wang Y , Waugh RE , Wan J . Piezo1 regulates mechanotransductive release of ATP from human RBCs. Proc Natl Acad Sci USA. 2015;112(38):11783‐11788. 26351678 10.1073/pnas.1507309112 PMC4586865

[44] Mousawi F , Peng H , Li J , et al. Chemical activation of the Piezo1 channel drives mesenchymal stem cell migration via inducing ATP release and activation of P2 receptor purinergic signaling. Stem Cells. 2020;38(3):410‐421. 31746084 10.1002/stem.3114 PMC7064961

[45] Virtanen P , Gommers R , Oliphant TE , et al. Author Correction: SciPy 1.0: fundamental algorithms for scientific computing in Python. Nat Methods. 2020;352. 10.1038/s41592-020-0772-5 PMC7056641 32094914

[46] Morrill EE , Tulepbergenov AN , Stender CJ , Lamichhane R , Brown RJ , Lujan TJ . A validated software application to measure fiber organization in soft tissue. Biomech Model Mechanobiol. 2016;15(6):1467‐1478. 26946162 10.1007/s10237-016-0776-3 PMC5328598

[47] Bandi E , Bernareggi A , Grandolfo M , et al. Autocrine activation of nicotinic acetylcholine receptors contributes to Ca^2+^ spikes in mouse myotubes during myogenesis. J Physiol. 2005;568(1):171‐180. 10.1113/jphysiol.2005.091439 16037088 PMC1474771

[48] Giniatullin A , Petrov A , Giniatullin R . Action of hydrogen peroxide on synaptic transmission at the mouse neuromuscular junction. Neuroscience. 2019;399:135‐145. 30593920 10.1016/j.neuroscience.2018.12.027

[49] Bernareggi A , Ren E , Giniatullin A , et al. Adenosine promotes endplate nAChR channel activity in adult mouse skeletal muscle fibers via low affinity P1 receptors. Neuroscience. 2018;383:1‐11. 10.1016/j.neuroscience.2018.04.044 29733889

[50] Mukhutdinova KA , Kasimov MR , Giniatullin AR , Zakyrjanova GF , Petrov AM . 24S‐hydroxycholesterol suppresses neuromuscular transmission in SOD1(G93A) mice: a possible role of NO and lipid rafts. Mol Cell Neurosci. 2018;88:308‐318. 29550246 10.1016/j.mcn.2018.03.006

[51] Barstad JA , Lilleheil G . Transversaly cut diaphragm preparation from rat. An adjuvant tool in the study of the physiology and pbarmacology of the myoneural junction. Arch Int Pharmacodyn Ther. 1968;175(2):373‐390. 5702955

[52] Sokolova E , Grishin S , Shakirzyanova A , Talantova M , Giniatullin R . Distinct receptors and different transduction mechanisms for ATP and adenosine at the frog motor nerve endings. Eur J Neurosci. 2003;18(5):1254‐1264. 12956724 10.1046/j.1460-9568.2003.02835.x

