# Supplementary material for: “Time window” effect of Yoda1‐evoked Piezo1 channel activity during mouse skeletal muscle differentiation
Source: Acta Physiol (Oxf). 2021 Jun 22;233(4):e13702. doi: 10.1111/apha.13702 (PMC9286833; doi:10.1111/apha.13702)
Supplement: Supplementary file 1 — Fig S1‐S5 [file APHA-233-0-s001.pdf]

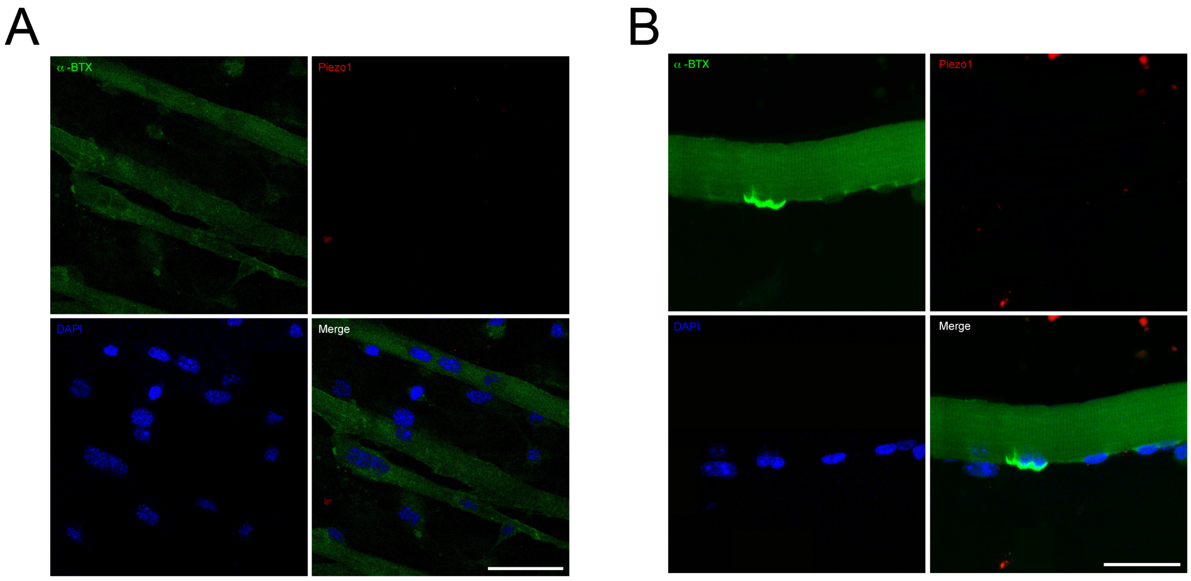

Fig. 1 SI Representative staining for Piezo1 and endplate in antigen retrieval experiments carried out in (A) 7 days differentiated myotubes and in (B) 24 h post-plating myofibers. Staining for Piezo 1 and endplate was performed following the procedure applied for control experiments using the primary Piezo 1 antibodies pre-absorbed overnight (at 4 °C) by a 10-fold excess of the specific blocking peptide (as indicated by manufacturer). Cells treated with the pre-absorbed primary antibody showed a marked reduction in Piezo1 fluorescence signal, while the endplate staining remained detectable. Scale bars: 25 μm in A; 20 μm in B. Experimental replicates n = 3; data from 3 independent experiments.

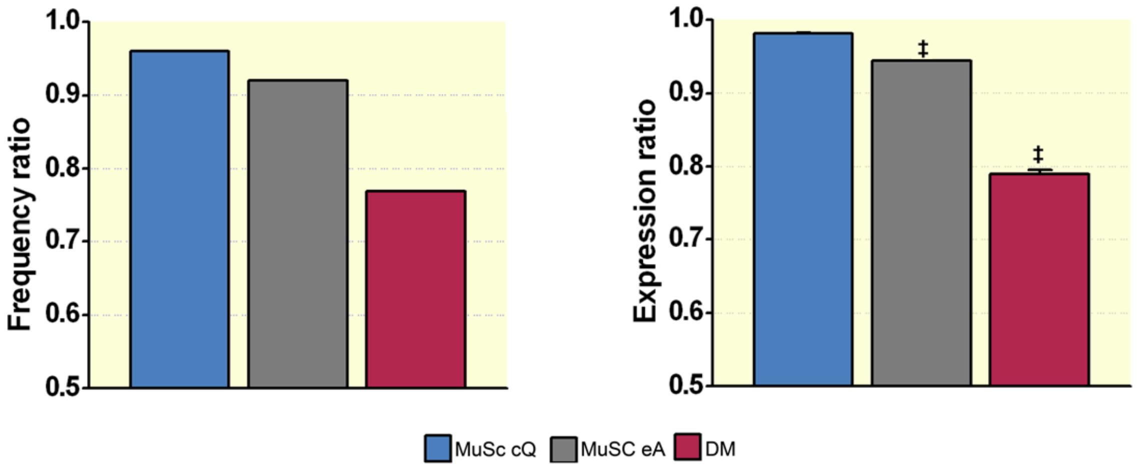

Fig. 2 SI. *Left*, the plot shows how frequently Piezo1 is expressed in the cell types of the dataset with respect the other genes. The frequency ratio represents the position of Piezo1 frequency with respect the other genes expressed in the same cells; ratio of 1 is assigned to the most frequently expressed gene in each cell type. Piezo1 results highly frequent in all the myogenic populations. In MuSc cQ, the frequency ratio is of 96.94% (n = 119), in MuSc eA 92.29% (n = 473) and in differentiated myocytes (DM) 77.38% (n = 183). *Right,* the plot shows the expression ratio of Piezo1 in the cell types of the dataset with respect the other genes. The expression ratio is the position of Piezo1 expression with respect the other genes, ratio of 1 is assigned to the most strongly expressed gene in the cell type.

Piezo1 gene is highly expressed in all the cell groups. In MuSc cQ the expression ratio is 98.10 ± 0.075% (n = 119), in MusSc eA 94.35 ± 1.03% (n = 473) and in differentiated myocytes (DM) 79.03 ± 5.12% (n = 183, $^{‡}P < 0.001$ Kruskall-Wallis test, Dunn's multiple comparison test).

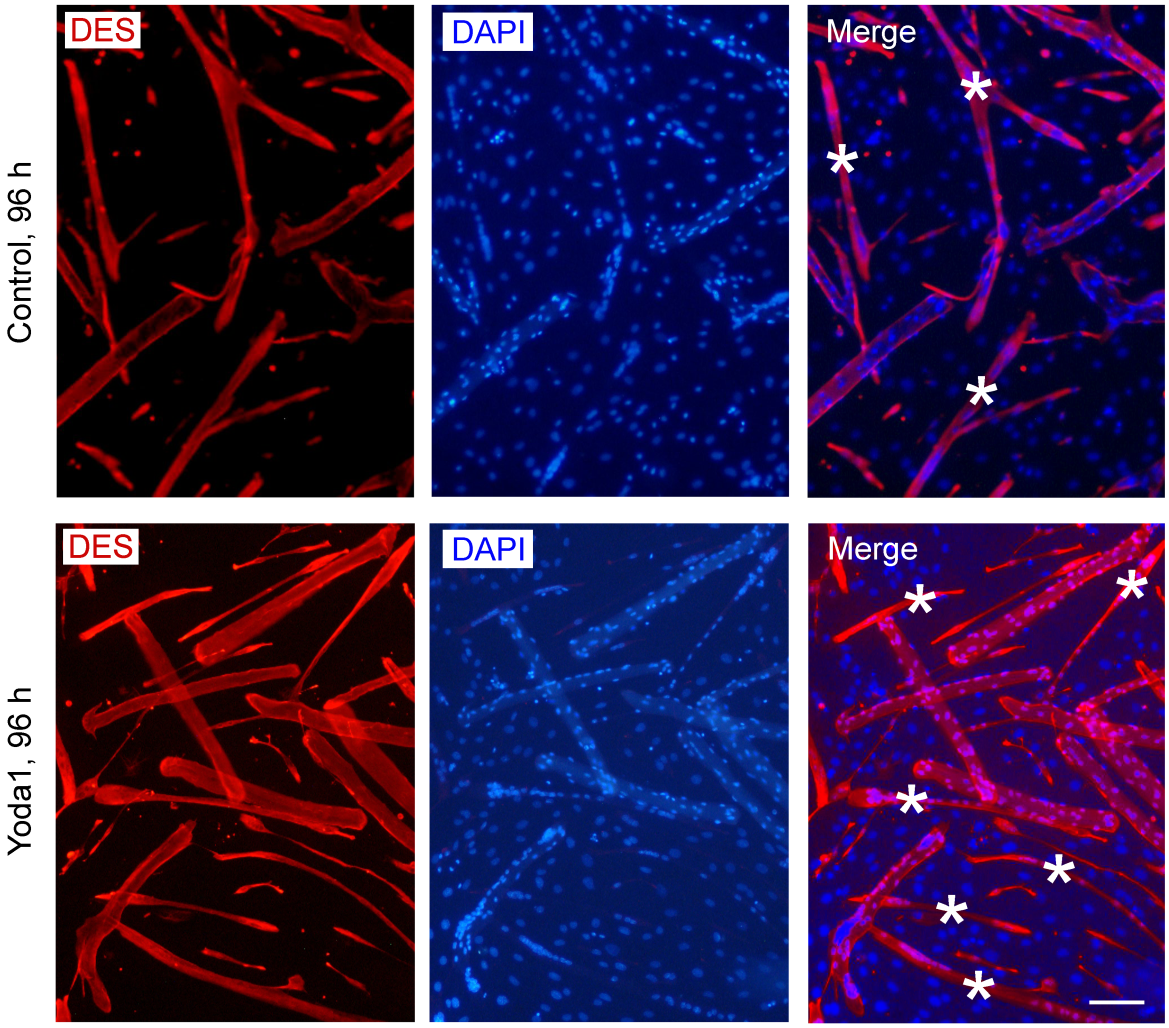

Fig. 3 SI. Myotubes in control and Yoda1-treated FDB-derived cultures. Representative desmin staining indicates the presence of myotubes (marked with asterisks) as revealed by centrally- located nuclei. Scale bar: 50 μm. Experimental replicates n = 3; data from 3 independent experiments.

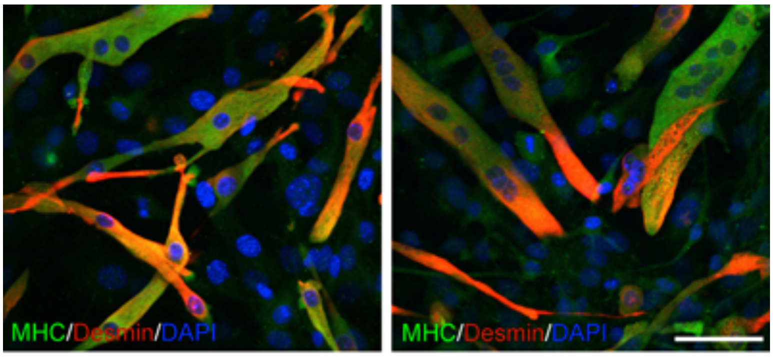

Fig. 4 SI. Yoda1 treatment did not impair myotube differentiation. Representative staining for Desmin and Myosine Heavy Chain (MHC) in myotubes of 10 days FDB-derived cultures in control conditions and in the presence of Yoda1 (3 μM). After dissociation the cells were treated with Yoda1 (3 μM) for 96 h in differentiation media, after that, Yoda 1 was washed out by replacing the cell culture media with fresh differentiation media. In both experimental conditions, all the myotubes co-expressed the two myogenic markers. Scale bar: 50 μm. Experimental replicates *n* = 3, data from 3 independent experiments.

.

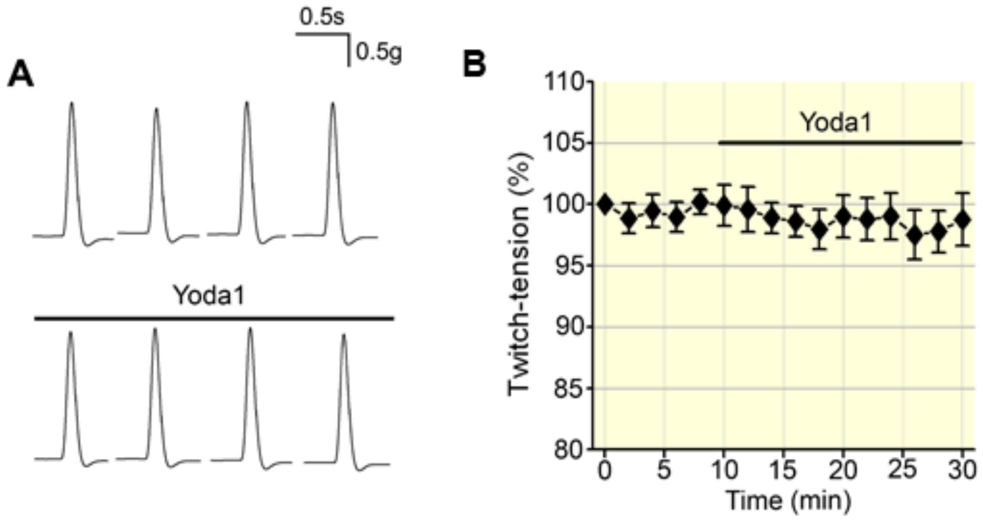

Fig. 5 SI. Effect of Yoda1 on mouse diaphragm twitch. A, Representative contraction traces in control conditions and after application of Yoda1 (5 μM). B, Time profile for Yoda1 effect on the amplitude of muscle contractions (*n* = 6 animals, P > 0.05, paired t - test).
